# Invasive Meningococcal Disease and COVID-19 Co-Infection: A Case Report

**DOI:** 10.7759/cureus.39713

**Published:** 2023-05-30

**Authors:** Carlos Espiche, Manuel Beltran, Yadanar Win Lei, Yennifer Gil Castano, Garry Francis-Morel, Michelle Dahdouh

**Affiliations:** 1 Internal Medicine, St. Barnabas Hospital (SBH) Health System and The City University of New York (CUNY) School of Medicine, Bronx, USA; 2 Internal Medicine, Medicine, St. Barnabas Hospital (SBH) Health System and The City University of New York (CUNY) School of Medicine, Bronx, USA; 3 Infectious Disease, St. Barnabas Hospital Health System, Bronx, USA

**Keywords:** covid-19 pandemic, covid 19, pulmonary critical care, pulmonary, meningoccocus

## Abstract

This case report presents a 53-year-old male patient infected with COVID-19 who developed acute respiratory distress syndrome (ARDS) and septic shock due to *meningococcemia*, despite the absence of clinical signs of meningitis. This patient's condition was complicated by pneumonia in the setting of myocardial failure. In the curse of the disease, it is remarked that the importance of early recognition of sepsis symptoms is crucial in distinguishing patients with COVID-19 from those with other infections and preventing fatal outcomes. The case presented an excellent opportunity to review meningococcal disease's intrinsic and extrinsic risk factors. With the identified risk factors, we propose different measures to be considered to diminish and recognize this fatal disease early.

## Introduction

*Neisseria meningitidis* is a gram-negative, aerobic diplococci bacterium that colonizes the non-ciliated columnar mucosal cells of the nasopharynx in humans and is transmitted through aerosols or secretions. The bacteria may cause systemic diseases by penetrating the mucosa and entering the bloodstream, and under certain circumstances, they may cross the blood-brain barrier and cause meningitis [1-3].

According to the 2020 National Notifiable Diseases Surveillance System of the Centers for Diseases Control and Prevention of the United States of America, there were 242 cases of meningococcal disease, resulting in an incidence of 0.08 per 100,000 population. Exposure to tobacco (active or passive), underlying immune defects (such as functional or anatomical asplenia, deficiency of properdin or terminal complement components), HIV infection, travel to a country with a hyperendemic or epidemic disease, household crowding, and viral upper respiratory infections are among several risk factors that may predispose individuals to invasive meningococcal infection [1,3,4]. Over the past two decades, there has been a decline in the incidence of invasive meningococcal disease (IMD) in the United States. This decrease can be attributed to increased vaccination rates, improved access to treatment, and routine post-exposure prophylaxis, which is nearly 95% effective [5]. Despite a reduction in meningococcal vaccine uptake, the incidence of IMD has declined during the COVID-19 pandemic due to the implementation of public health measures such as lockdowns, mask-wearing, and social distancing [2,6]. Although viral infections such as COVID-19 are well-known predisposing factors for bacterial infections, only a limited number of cases of coinfection with COVID-19 and meningococcal disease were reported during the early stages of the pandemic [7-10].

New York City (NYC), one of the most cosmopolitan cities in the world, has experienced a fluctuating prevalence of COVID-19, dropping from approximately 32% during the initial phases of the pandemic to an average of 23% following the implementation of vaccines and infection control measures [1]. In the past, NYC experienced two outbreaks of IMD between 2005 and 2006 and 2010 and 2013, primarily among the drug-using population, which necessitated extensive vaccination campaigns and treatment [4]. Due to the rapid spread of COVID-19, NYC became the first epicenter of the pandemic in the United States. Three years after the pandemic's onset and with a better understanding of COVID-19 infection, we present a rare case of coinfection with IMD and COVID-19.

## Case presentation

The following is a presentation of a case involving a 53-year-old male patient with controlled asthma who presented with abdominal pain, nausea, vomiting, and diarrhea for one day after consuming pizza. The patient had also received both doses of the COVID-19 vaccine. Upon admission, the patient was alert and experienced epigastric pain.

In the emergency department, the patient developed a spike in a fever of 100.5 F degrees and diarrhea. Physical examination revealed abdominal tenderness in the epigastric area without masses or organomegaly; bowel movements were present. There were no signs of rash or nuchal rigidity without focal deficits at the neurological exam. No edema in the legs or jugular vein distention. At chest auscultations, there was wheezing in both lungs and no intercostal retractions or secondary respiratory muscle usage at the beginning. The patient's oxygen saturation was 95% in room air with no respiratory distress. The patient was admitted with the diagnosis of enteritis and brought to the general medical floor. 

**Figure 1:**
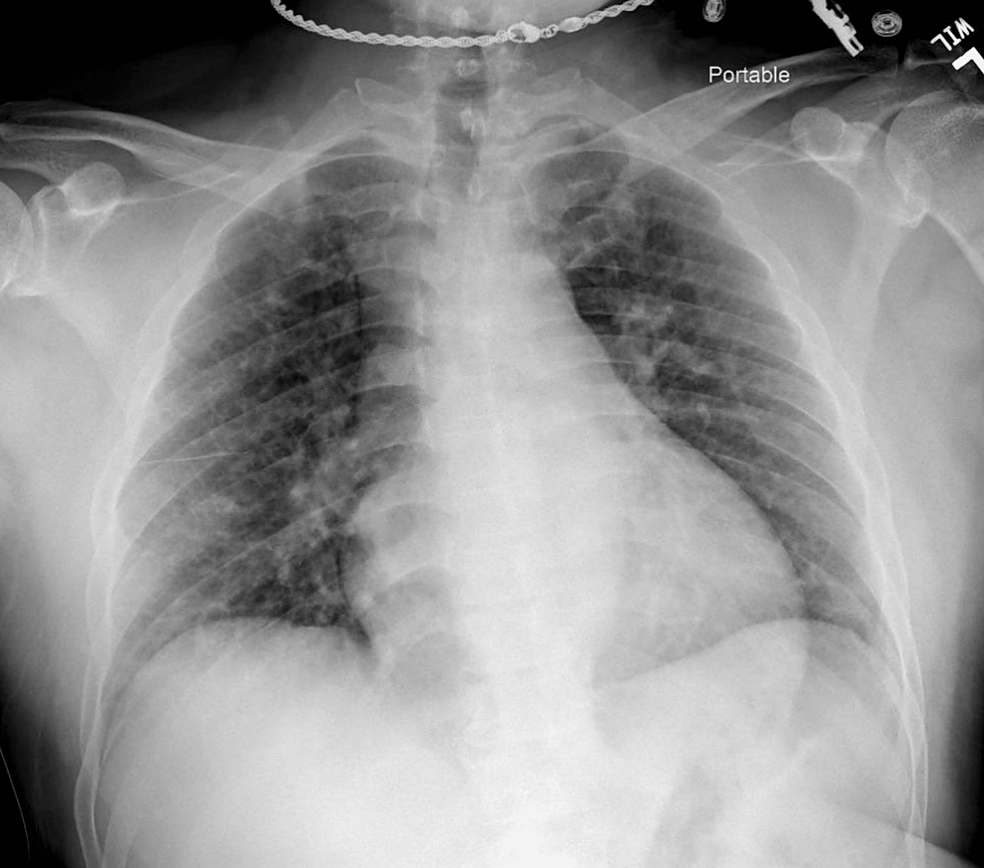
First chest radiography Posterior anterior chest radiography with preserved lung parenchyma and cardiac structure and unspecific anomalies. Showed no infiltrates or signs of effusion or pneumothorax.

The patient complains of mild shortness of breath on the general medical floor with 92%-93% oxygen saturation levels. After administering Duonebs 2.5 mg/3 ml as nebulization (albuterol-Ipatropium), the patient's saturation level increased to 98%. The results of the COVID-19 test were positive. Chest radiography showed no infiltrates, mild fibrosis, or signs of effusion or pneumothorax (Figure 1).

**Figure 2:**
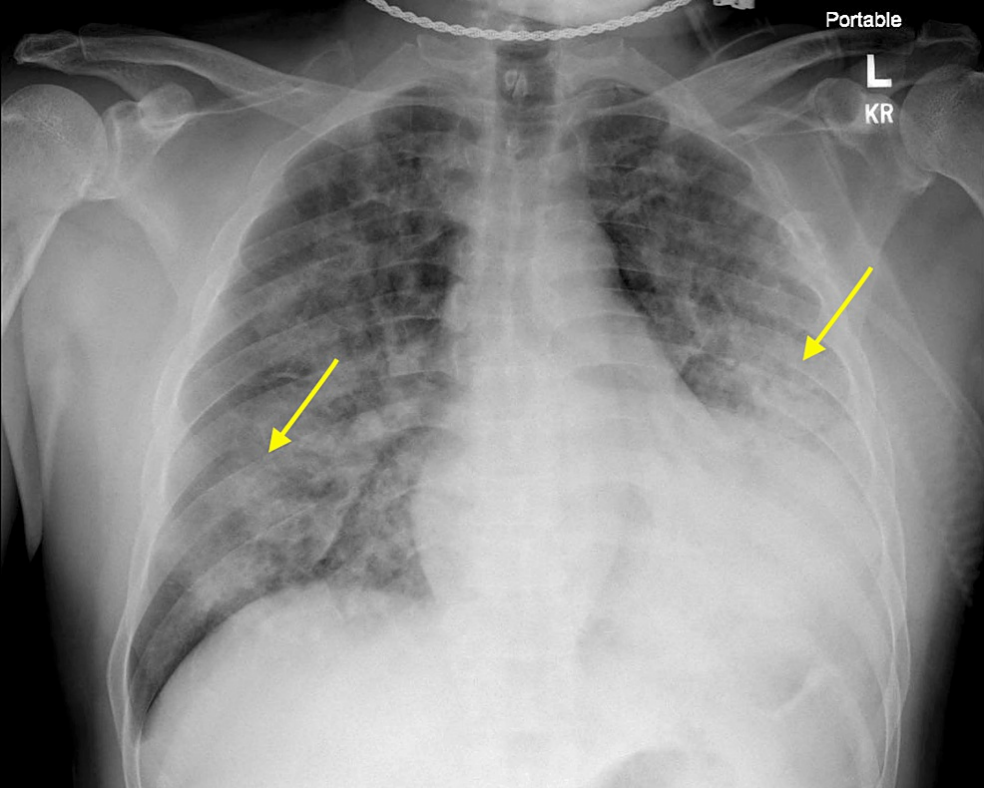
Chest radiography shows new consolidations Chest radiography revealed left lower lobe consolidation and bilateral patchy infiltrates.

The patient was placed in airborne contact isolation to prevent the spread of COVID-19 and started on remdesivir, vancomycin, meropenem, and azithromycin due to the concern of developing bacterial pneumonia associated with the viral infection. No systemic steroids were administered. However, the patient's oxygen saturation dropped to 85%, and tachypnea was observed at 28 respirations per minute. Their oxygen requirements increased over the following hours, and chest radiographs revealed left lower lobe consolidation and bilateral patchy infiltrates (Figure 2). 

The patient was transferred to the intensive care unit due to increased oxygen requirements and new radiological findings and was diagnosed with acute respiratory distress syndrome (ARDS) due to the new radiological findings associated with a paO2/FIO2 ratio of 147.5. The medical team placed the patient on a high-flow nasal cannula with a fraction of inspired oxygen (FiO2) of 100% and 60 L/min, which only achieved a saturation of 89% and a paO2/FIO2 ratio of less than 100. Steroids were added to the treatment regimen. Initially, the patient's condition improved using bilevel-positive airway pressure (BiPAP), but later, the patient's condition worsened, and the medical team started mechanical ventilation. The patient also had decreased blood pressure, which required vasopressors like norepinephrine at a maximum rate of 32 mcg/mL.

After intubation, blood cultures taken at admission were confirmed for gram-negative *coccobacilli*, specifically *Neisseria meningitidis*. Droplet precautions were initiated for 24 hours, and appropriate antibiotic therapy for meningococcal meningitis was administered. The patient received high doses of ceftriaxone at 2 grams every 12 hours intravenously and continued remdesivir and dexamethasone for severe COVID-19. A lumbar puncture and a brain-computer tomography were planned but not performed due to the patient's hemodynamic instability. Vancomycin, meropenem, and azithromycin were discontinued. The infection control team reported the case to the Department of Health (DOH) for chemoprophylaxis of close contacts. DOH identified the organism as serogroup C *Neisseria meningitidis* using a polymerase chain reaction.

Initial studies (Table 1) have demonstrated low levels of complement CH50 and C4, elevated levels of lactic acid, and procalcitonin levels exceeding one hundred. Abdominal computed tomography (CT) showed negative results, and echocardiography revealed severe global hypokinesis of the left ventricle with an estimated left ventricular ejection fraction (EF) of 20%-25%; the patient was not taking medications for this, and he had not been seen by a cardiologist before or had previous testing. Following catheterization, due to findings in the image testing that revealed nonstructural defects in the coronaries, it was suggested that the patient wear a life vest at discharge due to the high risk of ventricular arrhythmias.

**Table 1:** Relevant initial exams Relevant initial exams were included here with the study done, the values obtained, and the reference range.

Initial Exams	Value	Reference	Interpretation
Procalcitonin	>100.00	[0.00-0.08]	High
Lactic Acid	3.40	[0.50-2.20]	High
CH50	27.00	[>41.00]	Low
Complement C4	11.00	[12.00-38.00]	Low
Complement C3	88.00	[82.00-167.00]	Normal

Upon stabilization of the patient, a brain CT scan was performed, revealing the presence of congenital anomalies, specifically supratentorial ventriculomegaly, characterized by marked dilation of the lateral and third ventricles, as well as a cavum variant. Additionally, the scan exhibited diffuse hypoattenuation of the right cerebral hemisphere, with no apparent evidence of acute inflammatory processes or intracerebral hemorrhage. Following a four-day regimen of antibiotic therapy, the patient was cleared by neurology, and a subsequent lumbar puncture yielded negative results. The patient received a comprehensive treatment course, including seven days of ceftriaxone at 2 grams every 12 hours intravenous for meningococcemia, five days of remdesivir, and 10 days of dexamethasone treatment. Although the hospital course was complicated by methicillin-resistant *Staphylococcus aureus* (MRSA) pneumonia, successful extubation, and discharge were achieved. Table 2 shows the cerebrospinal fluid analysis.

**Table 2:** Cerebrospinal fluid analysis (CSF) Results of the cerebrospinal fluid analysis (CSF) after the lumbar puncture.

Parameter	Value/Description	Reference
Color	Colorless	Colorless
Appearance	Clear	Clear
Xanthochromia	Negative	Negative
WBC	4	[0-5/mm3]
RBC	475	[0-0/mm3]
Glucose	74	[40-70 mg/dL]
Protein Total	55	[15-45 mg/dL]

To prevent infections caused by the ACWY serogroup, the MenACWY meningococcal vaccine was administered to the patient at discharge, and HIV test results were negative at that time. Despite efforts by the cardiology department to provide outpatient follow-up care, the patient encountered challenges and missed most of their scheduled appointments.

## Discussion

Invasive meningococcal infection is influenced by intrinsic and extrinsic risk factors, such as underlying immune defects, deficiencies in complement components, viral infections, travel to hyperendemic regions, and household crowding [10]. The patient described in this case report had intrinsic risk factors, including a deficiency of terminal complement components. Terminal complement component deficiency is a known risk factor for meningococcal disease [11].

Unstable living conditions have been identified as an extrinsic risk factor for increased exposure to infectious diseases, including meningococcal disease. Homelessness, for instance, is associated with higher meningococcal disease risk due to shelter overcrowding, lower vaccination rates, and increased exposure to other infectious diseases [4,12].

Viral infections such as influenza A have been identified as risk factors for invasive meningococcal disease due to their ability to impair the immune response of the respiratory mucosa and promote bacterial virulence [2,10,13]. COVID-19 infection involves the activation of the lectin pathway and the spike and nucleocapsid proteins, which can directly activate the complement cascade via the lectin pathway, leading to complement consumption [14,15].

Although coinfection cases between N. meningitides and SARS-CoV-2 have been reported, a direct correlation between the two diseases has not been established [7,8,11,16]. However, meningococcal outbreaks have been linked to other respiratory viral infections, such as the 1918-1919 pandemic [17]. The initial presentation of coinfection may not be typical of COVID-19, and meningococcal pneumonia usually presents as lobar pneumonia, rapidly accompanied by ARDS.

The patient described in this case report did not exhibit typical symptoms of meningococcal diseases, such as vomiting, rash, or diarrhea, and meningeal presentation was unlikely. However, the lumbar puncture was performed days after antibiotic initiation. Guiddir et al. reported that early abdominal pain was the most frequent symptom in 105 cases of IMD, followed by gastroenteritis and diarrhea alone [18]. Atypical presentations can lead to delays in proper management and increased fatality rates. Therefore, prompt application of the sepsis protocol, including blood culture collection in the ED, was crucial for patient survival as it facilitated earlier identification and treatment of the organism.

It is important to note that after the COVID-19 pandemic, a wide variety of symptoms ranging from nonspecific general symptoms to hypoxia and gastrointestinal symptoms may raise suspicion of COVID-19 infection. COVID-19 testing has become mandatory in the emergency room before admission in most hospitals in heavily impacted cities such as NYC, resulting in the identification of most COVID-19 cases. However, this is not the case for other potentially fatal and highly contagious infections, such as IMD, which share several risk factors with COVID-19 and can be fatal if not properly treated.

## Conclusions

Household crowding and lack of vaccination are recognized as significant risk factors for meningococcal infection, which are also shared with COVID-19. As a result, conducting comprehensive interviews and physical examinations is critical. Early identification of sepsis symptoms in COVID-19 patients is crucial to differentiate them from other infections and prevent fatal outcomes.

This case report describes a unique instance of co-infection with COVID-19 and meningococcal disease in New York City, which has previously experienced outbreaks of invasive meningococcal disease among the drug-using population, necessitating widespread vaccination campaigns and treatment. The case underscores the connection between viral pandemics, such as COVID-19 or influenza, and uncommon bacteria, such as *meningococcus*. Timely detection and management of the case led to a positive outcome, underscoring the importance of early identification of *N. meningitidis*, a potentially lethal bacterium, in patients with similar risk factors. Recognition of the concomitant presentation of *N. meningitidis* with more prevalent infections such as COVID-19 is crucial.
